# *Vibrio cholerae* O1 with Reduced Susceptibility to Ciprofloxacin and Azithromycin Isolated from a Rural Coastal Area of Bangladesh

**DOI:** 10.3389/fmicb.2017.00252

**Published:** 2017-02-21

**Authors:** Shah M. Rashed, Nur A. Hasan, Munirul Alam, Abdus Sadique, Marzia Sultana, Md. Mozammel Hoq, R. Bradley Sack, Rita R. Colwell, Anwar Huq

**Affiliations:** ^1^Maryland Pathogen Research Institute, University of MarylandCollege Park, MD, USA; ^2^CosmosID, Inc.Rockville, MD, USA; ^3^Center of Bioinformatics and Computational Biology, University of Maryland Institute of Advanced Computer Studies, University of MarylandCollege Park, MD, USA; ^4^International Centre for Diarrhoeal Disease ResearchBangladesh, Dhaka, Bangladesh; ^5^Department of Microbiology, University of DhakaDhaka, Bangladesh; ^6^Johns Hopkins Bloomberg School of Public HealthBaltimore, MD, USA; ^7^Maryland Institute of Applied Environmental Health, University of MarylandCollege Park, MD, USA

**Keywords:** *Vibrio cholerae*, El Tor, antibiotic resistance, reduced susceptibility, ciprofloxacin, azithromycin

## Abstract

Cholera outbreaks occur each year in the remote coastal areas of Bangladesh and epidemiological surveillance and routine monitoring of cholera in these areas is challenging. In this study, a total of 97 *Vibrio cholerae* O1 isolates from Mathbaria, Bangladesh, collected during 2010 and 2014 were analyzed for phenotypic and genotypic traits, including antimicrobial susceptibility. Of the 97 isolates, 95 possessed CTX-phage mediated genes, *ctxA, ace*, and *zot*, and two lacked the cholera toxin gene, *ctxA*. Also both CTX^+^ and CTX^−^ *V. cholerae* O1 isolated in this study carried *rtxC, tcpA*^ET^, and *hlyA*. The classical cholera toxin gene, *ctxB1*, was detected in 87 isolates, while eight had *ctxB7*. Of 95 CTX^+^ *V. cholerae* O1, 90 contained *rstR*^ET^ and 5 had *rstR*^CL^. All isolates, except two, contained SXT related integrase *intSXT*. Resistance to penicillin, streptomycin, nalidixic acid, sulfamethoxazole-trimethoprim, erythromycin, and tetracycline varied between the years of study period. Most importantly, 93% of the *V. cholerae* O1 were multidrug resistant. Six different resistance profiles were observed, with resistance to streptomycin, nalidixic acid, tetracycline, and sulfamethoxazole-trimethoprim predominant every year. Ciprofloxacin and azithromycin MIC were 0.003–0.75 and 0.19–2.00 μg/ml, respectively, indicating reduced susceptibility to these antibiotics. Sixteen of the *V. cholerae* O1 isolates showed higher MIC for azithromycin (≥0.5 μg/ml) and were further examined for 10 macrolide resistance genes, *erm*(A), *erm*(B), *erm*(C), *ere*(A), *ere*(B), *mph*(A), *mph*(B), *mph*(D), *mef*(A), and *msr*(A) with none testing positive for the macrolide resistance genes.

## Introduction

*Vibrio cholerae*, the causative agent of cholera, is autochthonous to the estuarine and marine environment worldwide. Of more than 200 O-antigen serogroups identified in *V. cholerae*, only toxigenic O1 and O139 are primarily associated with epidemics and pandemics (Sack et al., 2004). Cholera, an ancient diarrheal disease, continues to be a serious threat in countries of Asia, Africa, and South America. Even though cholera is underreported in many countries, 3–5 million cases are recorded annually in different parts of the world, with a significant number of deaths (Ali et al., 2012). The case fatality rate of cholera has been reduced over the past few decades, mainly because patients are treated with oral and/or intravenous rehydration therapy together with appropriate dosage of antibiotics. Effective antibiotic treatment shortens the duration of diarrhea and limits the loss of body fluids by ca. 50% (Sack et al., 2004). However, antibiotic resistant enteropathogens, including *V. cholerae*, are emerging rapidly due to the selective pressure of antibiotics existing in the environment and from excessive use (Laxminarayan et al., 2013; Andersson and Hughes, 2014). *V. cholerae*, both O1 and O139, have developed resistance to several antimicrobial drugs, including tetracycline (TE), chloramphenicol (C), furazolidone, ampicillin (AM), and trimethoprim-cotrimoxazole, used successfully to treat cholera over the years (Garg et al., 2001; Kitaoka et al., 2011). As a consequence, multidrug resistant (MDR) *V. cholerae* has been on the rise, causing clinicians to face a serious challenge when deciding a drug of choice and regimen for treating cholera patients.

Two biotypes of *V. cholerae* O1, Classical (CL) and El Tor (ET) are universally recognized, with each possessing distinct phenotypic and genetic traits, including major virulence genes, i.e., toxin coregulated pilus (*tcpA*) and B-subunit of cholera toxin (*ctxB;* Kaper et al., 1995; Safa et al., 2010). Of the two biotypes, CL is associated with the sixth and presumably the earlier pandemics of cholera that occurred between 1817 and 1923 (Kaper et al., 1995; Devault et al., 2014), while ET is reported to have initiated the ongoing seventh pandemic in the early 1960s, gradually displacing the CL biotype (Kaper et al., 1995; Kim et al., 2015). Over the past two decades, variants of ET with only a few CL attributes (phage-encoded repressor *rstR*^CL^ and B-subunit of cholera toxin *ctxB*^CL^) have emerged in Asia and Africa. These variants are collectively known as atypical ET (Safa et al., 2010; Kim et al., 2015). Moreover, based on amino acid substitutions in CtxB, 12 different *ctxB* genotypes have been identified in *V. cholerae* (Kim et al., 2015). In 1992, *V. cholerae* O139 carrying the SXT/R391 family integrative conjugative element (ICE) appeared transiently as the major cause of cholera in Bangladesh and India (Albert et al., 1993; Ramamurthy et al., 1993; Waldor et al., 1996). SXT/R391 ICE was the first MDR marker detected in *V. cholerae*, conferring resistance to streptomycin (S), sulfamethoxazole, and trimethoprim (Waldor et al., 1996; Hochhut et al., 2001). SXT/R391 ICE also found to provide a selective advantage to *V. cholerae* O1 ET, a strain that has been tracked globally in three overlapping waves during the seventh pandemic (Mutreja et al., 2011).

Interestingly, outbreaks of cholera that occur in the coastal areas are seasonal each year in Bangladesh. For example, in Mathbaria, cholera occurs predominantly during the spring, months of March through May, with inhabitants lacking safe drinking water are most susceptible (Emch et al., 2008; Akanda et al., 2013). Several antibiotics are used to treat cholera, including doxycycline, ciprofloxacin (CIP), and azithromycin (AZ), all greatly influenced by the drug sensitivity pattern of the bacterium reported in the contemporary literature (Harris et al., 2012). In Bangladesh, a single dose of AZ or CIP currently is used for prophylactic treatment. Not surprising, *V. cholerae* O1 is now reported to have reduced susceptibility to CIP in Bangladesh, India, Vietnam, Haiti, Zimbabwe, and Western Africa (Islam et al., 2009; Quilici et al., 2010; Sjölund-Karlsson et al., 2011; Tran et al., 2012; Kumar et al., 2014; Khan et al., 2015). MDR *V. cholerae* O1 resistant to TE, AM, S, sulfonamides, norfloxacin, gentamicin, furazolidone, kanamycin (K), sulfamethoxazole-trimethoprim (SXT), and erythromycin (E), is currently circulating in cholera endemic countries of Asia and Africa (Finch et al., 1988; Faruque et al., 2007; Jain et al., 2011; Rashed et al., 2012; Dixit et al., 2014). Furthermore, genes conferring resistance to CIP and AZ have been shown to be transferred to *V. cholerae* via plasmids, gene cassettes, and mobile genetic elements with horizontal gene transfer mechanisms in environmental reservoir implicated in transforming sensitive bacteria to resistant (Kitaoka et al., 2011). Considering these phenotypic and genetic modifications that are reported, a study of 97 *V. cholerae* O1 isolates was undertaken to determine the antibiotic resistance/susceptibility status of *V. cholerae* O1 isolated from environmental samples and cholera cases in cholera endemic Mathbaria, Bangladesh.

## Materials and methods

### Bacterial strains

In this study, a total of 97 *V. cholerae* O1 isolated from rectal swabs and surface water samples collected in the coastal villages of Mathbaria, Bangladesh, between June, 2010 and December, 2014, as a part of epidemiological surveillance conducted by the International Centre for Diarrheal Disease Research, Bangladesh (ICDDR,B) were analyzed for antibiotic susceptibility and genotypic traits. Mathbaria is geographically adjacent to the Bay of Bengal, located ~165 km south-west of Dhaka city. Clinical isolates (*n* = 52) were obtained from rectal swabs of suspected cholera patients seeking treatment at the local health center during the cholera peak and off-peak season. Environmental isolates (*n* = 45) were obtained from water and plankton samples collected periodically at six different ponds and a river in the same area where the clinical samples were collected. The clinical and environmental samples were collected, transported, and subjected to bacteriological analysis for *V. cholerae*, following standard procedures (Alam et al., 2006a; Huq et al., 2012). Isolation and identification were performed according to standard methods (Alam et al., 2006a,b; Huq et al., 2012). All samples were collected according to protocols approved by institutional review boards at the Johns Hopkins University, University of Maryland (College Park, MD, USA), and ICDDR,B. Informed consent was obtained from the patients, and parents or legal guardians of the children who participated in this study. Genomic DNA was prepared from the presumptively identified *V. cholerae* isolates using the boiling lysis method of Park et al. (2013) and *V. cholerae* species-specific *ompW* PCR was done to confirm identity of the isolates (Nandi et al., 2000).

### Serogrouping

The serogroups of the *V. cholerae* isolates were confirmed by a slide agglutination test using specific polyvalent antisera for *V. cholerae* O1 and O139. Isolates showing positive agglutination reaction with O1 antisera were tested further using a serotype-specific monoclonal antibody, i.e., Inaba and Ogawa (Alam et al., 2006b). The serogroups of these isolates were reconfirmed by multiplex PCR, targeting O1-(*wbe*) and O139-(*wbf*) specific O biosynthetic genes, together with the cholera toxin gene (*ctxA;* Hoshino et al., 1998).

### Antimicrobial susceptibility

Susceptibility to antimicrobials was determined by standard disc diffusion on Muller-Hinton agar (BD, USA) according to Clinical and Laboratory Standards Institute guidelines for *V. cholerae* (CLSI, 2010a) and *Enterobacteriaceae* (CLSI, 2010b). *Escherichia coli* ATCC 25922 was used as a control for antimicrobial susceptibility. All strains of *V. cholerae* were tested for resistance to AM (10 μg), CIP (5 μg), C (30 μg), E (15 μg), K (30 μg), S (10 μg), TE (30 μg), nalidixic acid (NA, 30 μg), penicillin (P, 10 μg), and SXT (23.75 and 1.25 μg, respectively) using commercially available discs (BD BBL Sensi-Disc). Minimum inhibitory concentrations (MIC) of CIP and AZ were determined using *E*-test strips (bioMérieux-USA), according to the manufacturer's instructions. Cut-off levels for assessing resistance were determined following the CLSI document M45 guidelines (CLSI, 2010b).

### PCR assay

PCR assays were carried out to detect genes encoding accessory cholera enterotoxin (*ace*), zonula occludens toxin (*zot*), hemolysin (*hlyA;* Rivera et al., 2001), SXT-related integrase (*int*SXT; Hochhut et al., 2001) and biotype-specific (ET and CL) toxin coregulated pilus (*tcpA;* Rivera et al., 2001), phage-encoded repressor (*rstR;* Kimsey et al., 1998), and repeat in toxin (*rtxC;* Chow et al., 2001) using primers and conditions described previously. Double mismatch amplification mutation assay (DMAMA)-PCR was performed to identify three genotypes of the cholera toxin gene, i.e., *ctxB1, ctxB3*, and *ctxB7*, based on nucleotide substitution at position 58, 115, and 203 (Naha et al., 2012). *V. cholerae* O1 strains O395 (CL), N16961 (ET), and 2010EL-1786 were used as control for the PCR analysis. *V. cholerae* O1 isolates showing MIC for AZ ≥ 0.5 μg/mL were analyzed further for the macrolide resistance genes: *erm*(A), *erm*(B), and *erm*(C), that encode methylase; *ere*(A) or *ere*(B) encoding esterases; *mph*(A), *mph*(B), and *mph*(D) encoding phosphotransferases; and *mef* (A) and *msr*(A) encoding efflux pumps (Phuc Nguyen et al., 2009).

## Results

### Phenotypic and geneotypic characteristics

All 97 isolates produced colonies typical of *V. cholerae* on both taurocholate tellurite gelatin agar (TTGA) and thiosulfate citrate bile-salts sucrose (TCBS) agar. These isolates gave biochemical reactions characteristic of *V. cholerae* and reacted to polyvalent antibody specific for *V. cholerae* serogroup O1. Of 97 isolates, 89 gave positive agglutination with monovalent Ogawa antisera, while the remaining eight reacted positively with monovalent Inaba antisera. All isolates were serologically identified as *V. cholerae* O1. Notably, the serotype of 89 strains was determined to be Ogawa and eight to Inaba (Table 1).

**Table 1:** **Genetic characteristics and drug resistance of ***V. cholerae*** O1 isolated in Bangladesh**.

**Year of isolation**	**Number of strains**	**Source**	**Serotype**	***wbeO1***	***ctxA***	***ace***	***zot***	***tcpA***	***rtxC***	***ctxB*** **type**	***rstR***	***hlyA***	***int*****_SXT_**	**Drug resistance profile**
2010	7	Env	OGET	+	+	+	+	ET	+	*B1*	ET	+	+	S, NA, TE, SXT
	3	Env	OGET	+	+	+	+	ET	+	*B7*	ET	+	+	S, NA, SXT
	1	Clinical	OGET	+	+	+	+	ET	+	*B1*	ET	+	+	S, NA, TE, SXT
2011	3	Env	OGET	+	+	+	+	ET	+	*B1*	ET	+	+	S, NA, TE, SXT
	1	Env	OGET	+	+	+	+	ET	+	*B1*	ET	+	+	S, NA, SXT
	11	Clinical	OGET	+	+	+	+	ET	+	*B1*	ET	+	+	S, NA, TE, SXT
	4	Clinical	OGET	+	+	+	+	ET	+	*B7*	ET	+	+	S, NA, SXT
	1	Clinical	OGET	+	+	+	+	ET	+	*B1*	ET	+	+	S, NA, TE, E, SXT
2012	5	Env	OGET	+	+	+	+	ET	+	*B1*	ET	+	+	S, NA, TE, SXT
	3	Env	INET	+	+	+	+	ET	+	*B1*	CL	+	+	S, SXT
	1	Env	INET	+	+	+	+	ET	+	*B1*	ET	+	+	S, NA, SXT
	4	Clinical	OGET	+	+	+	+	ET	+	*B1*	ET	+	+	S, NA, TE, SXT
	2	Clinical	INET	+	+	+	+	ET	+	*B1*	CL	+	+	S, SXT
	1	Clinical	INET	+	+	+	+	ET	+	*B1*	ET	+	+	S, NA, TE, SXT
	1	Clinical	INET	+	+	+	+	ET	+	*B1*	ET	+	+	S, NA, SXT
	1	Clinical	OGET	+	+	+	+	ET	+	*B7*	ET	+	+	S, NA, SXT
	1	Clinical	OGET	+	+	+	+	ET	+	*B1*	ET	+	+	P, S, NA, TE, SXT
2013	10	Env	OGET	+	+	+	+	ET	+	*B1*	ET	+	+	S, NA, TE, SXT
	2	Env	OGET	+	+	+	+	ET	+	*B1*	ET	+	+	P, S, NA, TE, SXT
	10	Clinical	OGET	+	+	+	+	ET	+	*B1*	ET	+	+	S, NA, TE, SXT
	1	Clinical	OGET	+	+	+	+	ET	+	*B1*	ET	+	+	P, S, NA, TE, SXT
	1	Clinical	OGET	+	+	+	+	ET	+	*B1*	ET	+	−	NA
2014	9	Env	OGET	+	+	+	+	ET	+	*B1*	ET	+	+	S, NA, TE, SXT
	1	Env	OGET	+	+	+	+	ET	+	*B1*	ET	+	+	P, S, NA, TE, SXT
	10	Clinical	OGET	+	+	+	+	ET	+	*B1*	ET	+	+	S, NA, TE, SXT
	2	Clinical	OGET	+	−	−	−	ET	+	−	−	+	+	S, NA, TE, SXT
	1	Clinical	OGET	+	+	+	+	ET	+	*B1*	ET	+	−	NA

*Env, environmental; OGET, Ogawa El Tor; INET, Inaba El Tor; ET, El Tor; CL, classical*.

Genomic DNA of all isolates (*n* = 97) amplified *V. cholerae* species-specific genes, namely *ompW* and O-antigen biosynthetic-*wbe* (O1) confirming identification as *V. cholerae* O1. None amplified the O-antigen biosynthetic-*wbf* (O139). As shown in Table 1, except for two isolates, all amplified the CTX-phage mediated genes *ctxA, ace*, and *zot*, suggesting 95 of the isolates were toxigenic *V. cholerae* O1. The PCR assay results also showed *hlyA* gene present in all isolates (Table 1). Of 97 *V. cholerae* O1, *intSXT* was identified in 95 isolates, while two lacked *intSXT*. All *V. cholerae* O1 isolates contained the ET biotype specific *tcpA* and *rtxC*, reflecting ET attributes. Among the 95 toxigenic *V. cholerae* O1 isolates, 90 possessed *rstR* of the ET biotype (*rstR*^ET^), while the remaining five revealed CL biotype specific *rstR*^CL^. Unlike hybrid ET strains, none of the toxigenic isolates contained both *rstR*^ET^ and *rstR*^CL^. DMAMA-PCR detected cholera toxin gene of CL biotype (*ctxB*1) in 87 *V. cholerae* O1 isolates, while 8 had Orissa variant or Haiti variant cholera toxin (*ctxB*7; Table 1). Overall, the PCR results confirmed that 90 of the *V. cholerae* O1 isolates were atypical ET, possessing the *rstR*^ET^ and either *ctxB1* or *ctxB7* gene. Five toxigenic *V. cholerae* O1 possessing *rstR*^CL^ and *ctxB1* are designated as variant ET and their genetic attributes were similar to the Matlab variant (MJ1236) isolated in 1994 in Matlab, Bangladesh. As shown in Table 1, *V. cholerae* O1 variant ET was isolated from both clinical and environmental sources in Mathbaria, Bangladesh only in 2012. *V. cholerae* O1 atypical ET was associated with cholera cases that occurred during June, 2010, and December, 2014, in Mathbaria, Bangladesh and these strains were also isolated frequently from environmental sources (Table 1).

**Figure 1:**
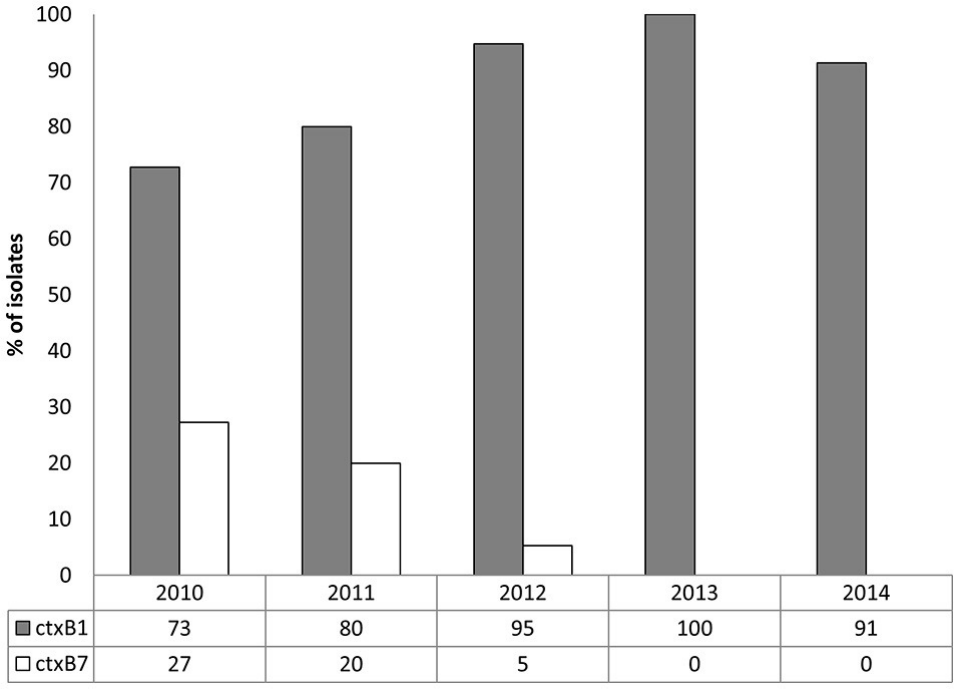
**Distribution of ***ctxB*** genotypes in 97 ***V. cholerae*** O1 from Mathbaria, Bangladesh isolated during 2010 and 2014**. In both the clinical and environmental isolates, *ctxB1* (gray bars) was predominant in each year. Although *ctxB7* in the *V. cholerae* O1 isolates (white bars) was detected in relatively low percentage in 2010, 2011, and 2012, it was not detected in 2013 and 2014.

As shown in Figure 1, the CL type cholera toxin genotype, *ctxB1*, was predominant, having been detected in 73, 80, 95, 100, and 91% *V. cholerae* O1 isolates in 2010, 2011, 2012, 2013, and 2014, respectively. In contrast, Orissa, or Haiti variant cholera toxin genotype *ctxB7* was found in 27, 20, and 5% isolates in 2010, 2011, and 2012, respectively. Remarkably, *ctxB7* was not detected in *V. cholerae* O1 isolated in 2013 and thereafter (Table 1). Although, 9% of the *V. cholerae* O1 were non-toxigenic in 2014, *ctxB1* was the only genotype prevailed among toxigenic isolates in 2013 and 2014.

### Antimicrobial susceptibility

**Figure 2:**
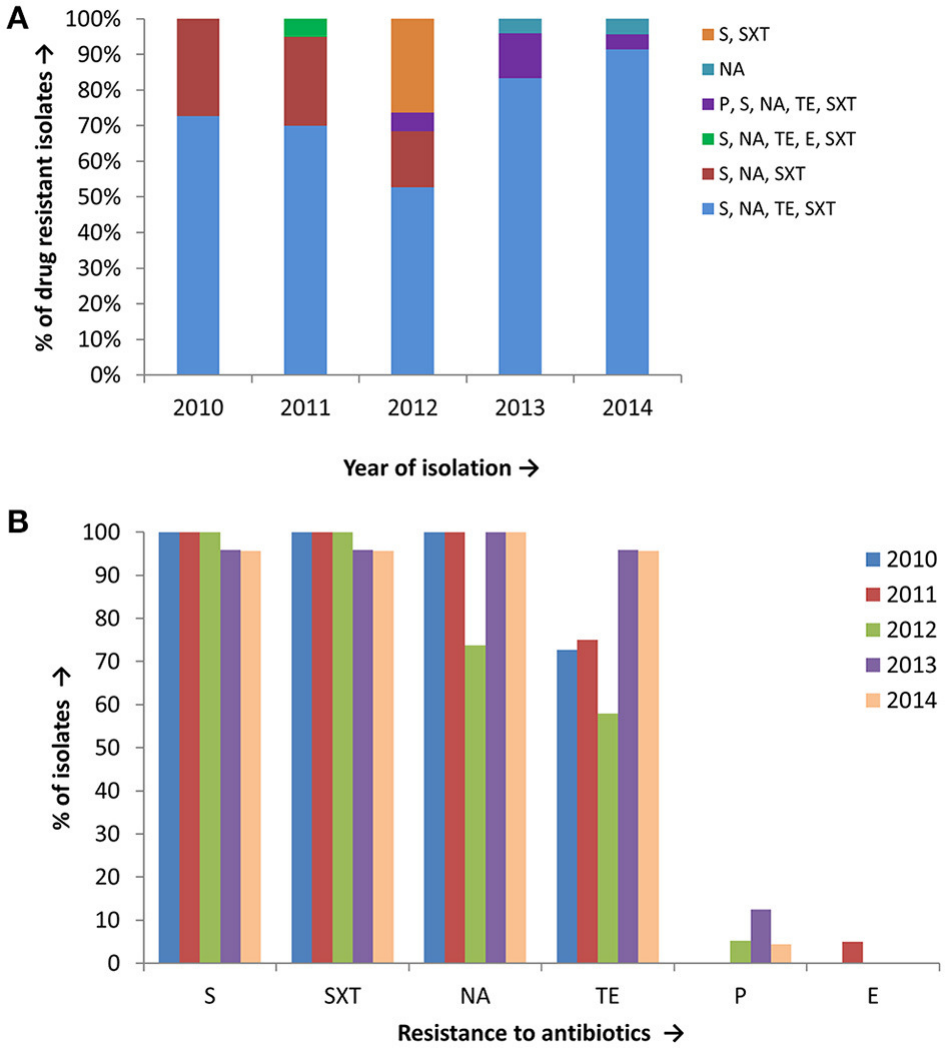
**(A)** Drug resistance profile of 97 *V. cholerae* O1 isolates from Mathbaria, Bangladesh. Six different resistance profiles were observed for *V. cholerae* O1 isolated during 2010 and 2014, of which four profiles were multidrug resistance: resistance to S, NA, TE, and SXT was most abundant and predominant in *V. cholerae* O1 **(B)** *V. cholerae* O1 isolated during 2010 and 2014 showing resistance to six different drugs. The majority of the isolates were resistant to S, SXT, TE, and NA, while only a few were resistant to P and E.

Antimicrobial susceptibility tests, using ten different antibiotics revealed that 93% of the total set of *V. cholerae* O1 isolates were MDR, i.e., resistant to at least three different antibiotics drugs (Table 1). As shown in Figure 2A, six different resistance profiles were observed, with a range of resistance to one to five antibiotics during 2010 and 2014. *V. cholerae* O1 showing resistance to S, NA, TE, and SXT was the dominant pattern (53–91%) each year between 2010 and 2014 (Figure 2A). Interestingly, resistance of *V. cholerae* O1 to P, S, NA, SXT, E, and TE varied during the years of the study period. As shown in Figure 2B, 100% of the *V. cholerae* O1 showed resistance to S and SXT in 2010, 2011, and 2012. However, S and SXT resistance fell to 96% the following 2 years, 2013 and 2014. The SXT-related integrase (*intSXT*) was detected in all isolates resistant to S and SXT, suggesting the SXT/R391 ICE mediated the resistance to S and SXT. Except for five of the *V. cholerae* O1 variant ET isolated in 2012, all were resistant to NA (Figure 2B), an indicator of reduced susceptibility to CIP. TE resistant *V. cholerae* O1 comprised 73, 75, 58, 96, and 96% in 2010, 2011, 2012, 2013, and 2014, respectively (Figure 2B). Of 97 *V. cholerae* O1, five showed resistance to P during 2012 and 2014, while only one isolate showed resistance to E in 2011. Notably, all 97 *V. cholerae* O1 isolates were uniformly sensitivity to AM, CIP, C, and K.

### MIC of ciprofloxacin

**Figure 3:**
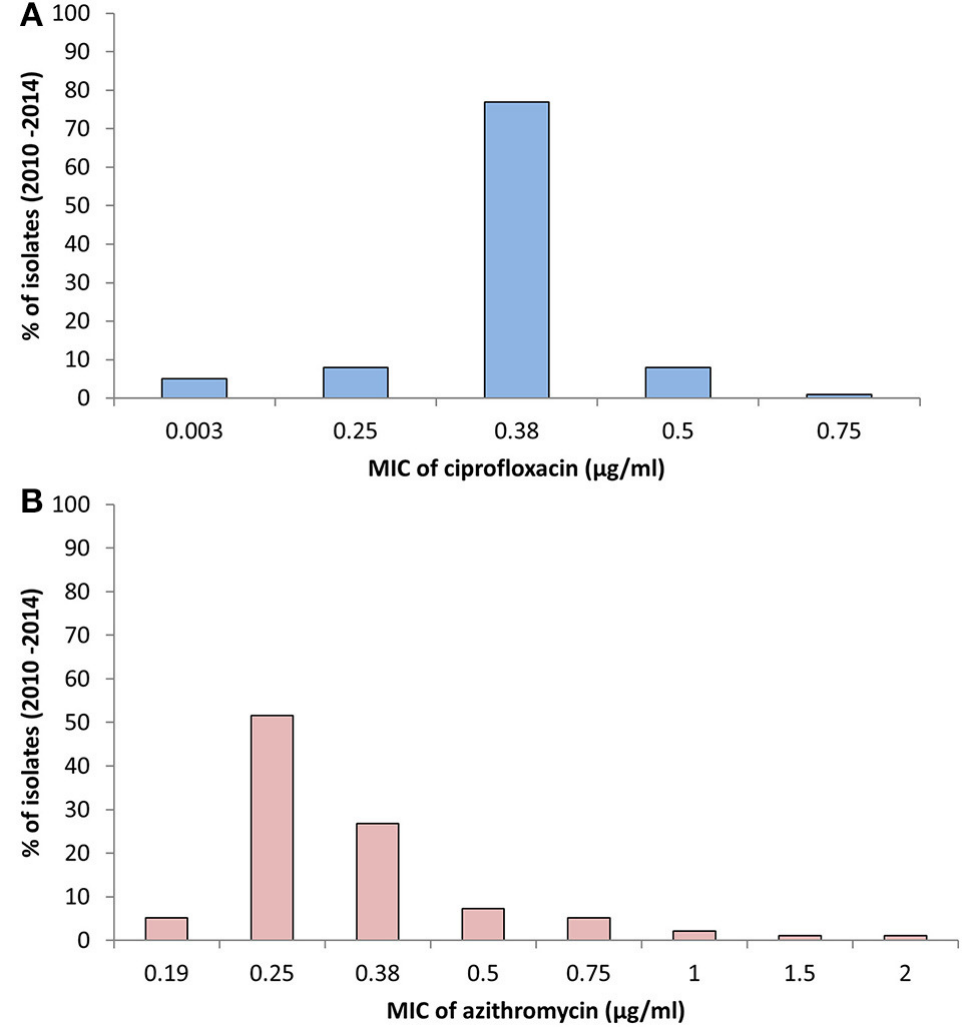
**(A)** MIC of CIP for 97 *V. cholerae* O1 isolates from Mathbaria, Bangladesh. The MIC range for CIP was 0.003–0.75 μg/ml. The majority of the isolates showed MIC 0.38 μg/ml, while a low percentage had MIC of 0.5 and 0.75 μg/ml. **(B)** MIC of AZ for 97 *V. cholerae* O1 isolates from Mathbaria, Bangladesh. The range of MIC for AZ was 0.19–2 μg/ml. A relatively low percent of the isolates had a MIC of 2 μg/ml, the sensitivity-borderline for *V. cholerae*.

The MIC of CIP of all 97 *V. cholerae* O1 isolates was determined to be 0.003–0.75 μg/ml during 2010 and 2014. As shown in Table 2, MIC_50_ and MIC_90_ of CIP was 0.5 μg/ml in 2010. However, the MIC_50_ and MIC_90_ were 0.38 μg/ml in 2011, maintained consistently over the following years until 2014. Five of the *V. cholerae* O1 variant ET isolated in 2012 had an MIC for CIP of 0.003 μg/ml. Only 1% of the total set of strains had the highest MIC, 0.75 μg/ml, while 77% had an MIC of 0.38 μg/ml (Figure 3A).

**Table 2:** **Minimum inhibitory concentration of ciprofloxacin and azithromycin for 97 ***V. cholerae*** O1 isolates**.

**Year of isolation**	**No. of strains**	**Ciprofloxacin**			**Azithromycin**		
		**MIC Range (μg/ml)**	**MIC_50_**	**MIC_90_**	**MIC Range (μg/ml)**	**MIC_50_**	**MIC_90_**
2010	11	0.38–0.75	0.5	0.5	0.19–0.75	0.25	0.38
2011	20	0.25–0.5	0.38	0.38	0.25–1.5	0.25	0.5
2012	19	0.003–0.38	0.38	0.38	0.25–0.75	0.38	0.75
2013	24	0.25–0.38	0.38	0.38	0.25–1	0.25	0.38
2014	23	0.25–0.38	0.38	0.38	0.19–2	0.25	0.5

### MIC of azithromycin

The MIC of AZ for 97 of the *V. cholerae* O1 isolates was 0.19–2.00 μg/ml. As shown in Table 2, except for the 2012 isolates, MIC_50_ was consistently 0.25 μg/ml. Year-wise data revealed that the lowest MIC_90_ was 0.38 μg/ml in 2010 and 2013 and increased to 0.5 and 0.75 μg/ml in 2011 and 2012, respectively (Table 2). As shown in Figure 3B, 52 and 27% of the total isolates had MIC 0.25 and 0.38 μg/ml, respectively. The highest MIC of 2.00 μg/ml occurred in 1% of the *V. cholerae* O1 isolates (Figure 3B). Sixteen (16%) *V. cholerae* O1 with an MIC of ≥0.5 μg/ml were analyzed further for 10 macrolide resistance genes. PCR assay results revealed that none of the isolates contained the following macrolide resistance genes: *erm*(A); *erm*(B); *erm*(C); *ere*(A); *ere*(B); *mph*(A); *mph*(B); *mph*(D); *mef* (A); and *msr*(A).

## Discussion

Endemic cholera occurs in many geographic locations of Bangladesh each year, with two distinct seasonal peaks, one in the spring (March–May) and the other in the fall (September–November; Emch et al., 2008; Akanda et al., 2013). The Ganges delta region of the Bay of Bengal is a well-known reservoir of *V. cholerae* where it has established residence for centuries (Sack et al., 2004). Historically, this part of Asia has been affected severely by both CL and ET cholera during the seventh pandemic up to 1991, prior to the disappearance of CL strains from Bangladesh (Siddique and Cash, 2014). Epidemiological data suggest that most of the recorded epidemics struck the coastal populations first (Jutla et al., 2010), a pattern typical of recent epidemics in Bangladesh as well before reaching inland (Akanda et al., 2013). Since the beginning of the ongoing seventh pandemic, *V. cholerae* O1 strains have undergone multiple genetic changes, with the evolution of new clones and also atypical ET strains (Chun et al., 2009; Safa et al., 2010; Kim et al., 2014). Results of this study show association of *V. cholerae* O1 atypical ET with cholera occurring in the coastal areas of Mathbaria, Bangladesh during 2010 and 2014. Since 2001, atypical ET emerged as the major cause of cholera in Bangladesh superseding prototype ET, although isolated a decade earlier in the 1990s in Matlab, Bangladesh (Nair et al., 2006; Safa et al., 2006). This transition was considered remarkable for cholera epidemiology, mainly because atypical ET strains possessing CL cholera toxin (*ctxB1*) cause a more severe cholera than prototype ET (Siddique et al., 2010). In recent years, several non-synonymous mutations have been detected in the *ctxB* gene, although, correlation of these mutations with clinical outcomes of the disease remains to be clarified (Kim et al., 2015). The *ctxB7* genotypes have an amino acid substitution at position 20 [histidine (H)→asparagine (N)] first reported in a cholera outbreak in Orissa, India (Kumar et al., 2009). Later, *V. cholerae* O1 carrying *ctxB7* was determined to be associated with cholera in Haiti, Zimbabwe, India, Bangladesh, Nepal, Nigeria, and Cameroon (Quilici et al., 2010; Chin et al., 2011; Hasan et al., 2012; Rashed et al., 2012; Marin et al., 2013; Dixit et al., 2014). In Mathbaria, Bangladesh, *ctxB1* was consistently dominant to *ctxB7* during 2010 and 2012, whereas *ctxB7* was not detected thereafter. The alternating dominance of *ctxB1* and *ctxB7*, i.e., one genotype disappearing transiently for 2 or 3 years and reappearing in the following years with remarkable dominance, was previously observed in *V. cholerae* O1 causing cholera in Kolkata, India, and Dhaka, Bangladesh (Rashed et al., 2012; Mukhopadhyay et al., 2014; Rashid et al., 2016).

Bacterial resistance to antimicrobial drugs is a serious public health concern worldwide and antibiotic therapy constitutes a major component of the clinical management of cholera. An antimicrobial drug considered to be a successful therapeutic agent may not be successful in the future and notably so if *V. cholerae* acquires resistance to drugs of choice. Resistance can arise from single or multiple mutations in target genes or by acquisition of resistance genes carried by mobile genetic elements, such as plasmids, transposons, integrons, and ICEs (Kitaoka et al., 2011). Prior to the use of macrolides and fluoroquinolone drugs for treatment of cholera, TE was the drug of choice, except for young children and pregnant women (Greenough et al., 1964; Sack et al., 2004). However, tetracycline was limited as a drug of choice because of the emergence of resistant *V. cholerae* O1 to AMP, KN, S, and SXT, as well as TE, in Asia and Africa (Mhalu et al., 1979; Glass et al., 1980). In this study, 93% of the *V. cholerae* O1 strains tested proved to be multidrug resistant, mostly resistant to S, NA, TE, and SXT. Despite having spatio-temporal variation in the resistance profile, the multidrug resistant *V. cholerae* O1 was consistently identified as the etiological agent of cholera epidemics in Asia and Africa, and most recently in Haiti (Mhalu et al., 1979; Glass et al., 1980; Dalsgaard et al., 2001; Jain et al., 2011; Sjölund-Karlsson et al., 2011; Rashed et al., 2012; Tran et al., 2012). A recent study from China reported that the prevalence of multidrug resistant *V. cholerae* O1 strains has been increased rapidly since 1993, showing resistance to AMP, NA, TE, and SXT (Wang et al., 2012). The same study also revealed relatively low number of *V. cholerae* O1 has reduced susceptibility to azithromycin in China that were isolated only in 1965, 1998, and 2006 (Wang et al., 2012). Drug resistant markers, such as SXT ICE, class 1 integrons, and low molecular weight plasmids are commonly found in multidrug resistant *V. cholerae* O1 (Kitaoka et al., 2011). Interestingly, a recent study reported the presence of a transmissible multidrug resistant plasmid (IncA/C) in Haitian *V. cholerae* isolates possessing several multidrug resistance determinants, i.e., *aac(3)-IIa, bla*_CMY−2_, *bla*_CTX−M−2_, *bla*-_TEM−1_, *dfrA15, mphA, sul1, tetA, floR, strAB*, and *sul2* (Folster et al., 2014).

Among the fluoroquinolone antibiotics, only CIP has been recommended by the Pan American Health Organization and International Centre for Diarrhoeal Disease Research, Bangladesh for treatment of cholera. Although, *V. cholerae* O1 has not shown complete resistance to CIP, current epidemiological data confirm a gradual increase in the MIC of CIP has been occurring (Khan et al., 2015). *V. cholerae* O1 with reduced susceptibility to CIP has been reported in different parts of the world and appears to be disseminating globally (Islam et al., 2009; Quilici et al., 2010; Sjölund-Karlsson et al., 2011; Tran et al., 2012; Khan et al., 2015). A recent study showed that the MIC of CIP for *V. cholerae* O1 has increased 45-fold in a 19 year time-span in Bangladesh. That is, the MIC was 0.010 μg/ml in 1994 and has increased dramatically to 0.475 μg/ml in 2012 (Khan et al., 2015). In our study, 95% of the *V. cholerae* O1 isolated in Mathbaria, Bangladesh, showed reduced susceptibility to CIP during 2010 and 2014. Notably, the CIP MIC_50_ and MIC_90_ did not show rapid change in the 5 year of our study period and the MIC remained below the susceptibility breakpoint (≤ 1 μg/ml) according to CLSI guidelines (CLSI, 2010a). It is important to note that all *V. cholerae* O1 atypical ET isolates were resistant to NA, another drug in the quinolone group. *V. cholerae* O1 showing resistance to NA is an indicator of reduced susceptibility to CIP (Khan et al., 2015). The genetic basis of quinolone drug resistance in *V. cholerae* is the accumulation of mutations in *gyrA* (83_Ser → Ile_) and *parC* (85_Ser → Leu_) (Kitaoka et al., 2011). These point mutations have been detected in currently circulating *V. cholerae* O1 associated with cholera epidemics in Bangladesh, India, Nepal, Nigeria, Cameroon, and Haiti (Quilici et al., 2010; Sjölund-Karlsson et al., 2011; Hasan et al., 2012; Dixit et al., 2014).

Frequent use of a specific group of antibiotics for treatment of cholera over a prolonged period will increase the likelihood of bacterial resistance. Global dissemination of *V. cholerae* O1 with reduced CIP sensitivity raises a serious concern for clinical management of cholera in countries where the disease is endemic. Results of a recent study showed that single-dose CIP used to treat cholera was not as effective as it was in the past because of the emergence of *V. cholerae* O1 less susceptible to CIP and NA (Khan et al., 2015). Single dose AZ has been introduced as an alternative treatment for cholera in India and Bangladesh. However, the sensitivity breakpoint guidelines for the AZ disc diffusion assay has not yet been published by the CLSI for *V. cholerae* (CLSI, 2010a). In this study, all *V. cholerae* O1 isolates showed a reduced susceptibility to AZ and the MIC for 1% of the isolates was at the sensitivity breakpoint borderline (≤2 μg/ml). Interestingly, none of the *V. cholerae* O1 (AZ MIC ≥ 0.5 μg/ml) possessed macrolide resistance genes that have been reported for the *Enterobacteriaceae* (Phuc Nguyen et al., 2009). Although at a relatively low incidence, E and AZ resistant *V. cholerae* O1 have been reported in Bangladesh and India (Faruque et al., 2007; Bhattacharya et al., 2012).

Reduced susceptibility to CIP and AZ is alarming for cholera-endemic countries of Asia and Africa. Environmental factors trigger seasonal cholera in endemic countries including Bangladesh, but cholera cases have occurred in other countries immediately after a devastating natural calamity, e.g., floods, earthquakes, typhoons, and cyclones. The morbidity and mortality rates of cholera, which were under control for several decades, can be expected to increase if *V. cholerae* O1 acquires full resistance to currently used drugs. Considering the global burden of cholera, it is important that the appropriate antibiotic and appropriate concentration be used to treat cholera. Indiscriminate use of antibiotics, for example in agriculture and animal husbandry for disease management should be controlled to assure continued success of antibiotic for the treatment of disease in humans, including cholera. Therefore, global monitoring of antimicrobial sensitivity of *V. cholerae* O1 is essential to assess clinical efficacy of drugs worldwide.

## Author contributions

SR and AH contributed to the design of the study. SR also performed all research works in the laboratory, analyzed data, and wrote the manuscript. AS and MS collected clinical and environmental samples from the field area and processed all samples at ICDDR,B. NH, MA, MH, RS, RC, and AH contributed to revising the manuscript critically for important intellectual content. All authors discussed, read, and approved the final manuscript.

## Funding

This research was supported by National Institute of Allergy and Infectious Disease (NIAID) grant no. 2RO1A1039129-11A2 from the National Institutes of Health (NIH).

### Conflict of interest statement

The authors declare that the research was conducted in the absence of any commercial or financial relationships that could be construed as a potential conflict of interest.
